# Perceived usefulness of a mnemonic to improve nurses’ evaluation of reported penicillin allergies

**DOI:** 10.1017/ash.2023.177

**Published:** 2023-07-11

**Authors:** Eileen J. Carter, Carol Schramm, Kate Baron, Meagan M. Zolla, Katherine Zavez, David B. Banach

**Affiliations:** 1 University of Connecticut School of Nursing, Storrs, Connecticut; 2 UConn John Dempsey Hospital, Farmington, Connecticut; 3 Department of Statistics, University of Connecticut, Storrs, CT; 4 University of Connecticut School of Medicine, Farmington, Connecticut; 5 Yale School of Public Health, New Haven, Connecticut

## Abstract

We surveyed clinicians to evaluate the perceived usefulness of a mnemonic, STORY, to improve penicillin allergy evaluation. Survey responses indicated that the perceived usefulness of STORY was high, and support for nurses’ involvement in penicillin allergy assessment was high. Future research may evaluate the feasibility of STORY implementation in clinical care.

An estimated 32 million individuals in the United States have a reported allergy to penicillin, yet >90% of those with reported penicillin allergies who undergo testing do not have a true penicillin allergy.^1^ Erroneously reported penicillin allergies mislead antibiotic selection and increase patient’s risk for drug-resistant infections and adverse events.^2^ Clinical histories are essential to risk-stratifying patients reporting a penicillin allergy,^1^ but the documentation of penicillin allergies is commonly incomplete, impeding risk stratification and perpetuating penicillin avoidance. In a large analysis of the records of 5,023 penicillin adverse drug reactions across 3 Australian hospitals, reaction descriptions were absent in 21% of records.^3^ Nurses entered most (58%) of the records and were more likely to include a description of the reaction when compared to medical personnel.^3^

In 2019, the Centers for Disease Control and Prevention (CDC) called upon nurses to improve the comprehensive evaluation of penicillin allergies as part of hospital antibiotic stewardship programs.^4^ A multisite qualitative study found that nurses are motivated to comprehensively evaluate their patient’s reported penicillin allergy but are unaware of the questions to ask when conducting a penicillin allergy history.^5^ We developed a mnemonic, STORY, to facilitate nurses’ recall of the questions to ask when taking a penicillin allergy history (Fig. 1). STORY frames the assessment as a narrative account of the alleged allergy. Each letter of STORY represents an element of the penicillin allergy assessment that informs risk stratification.^1^ We evaluated the perceived usefulness of STORY among clinicians.

**Figure 1.**
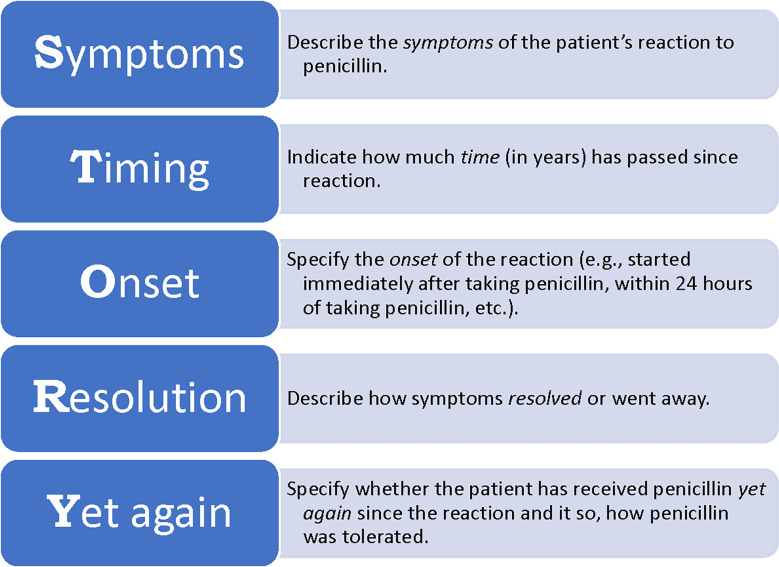
STORY mnemonic to facilitate the comprehensive evaluation of reported penicillin allergies.

## Methods

This cross-sectional survey was conducted among practicing nurses and prescribers working in the surgical areas of a medium-size academic medical center in the northeastern United States. To assess the perceived usefulness of STORY, we adapted a prior survey that evaluated the perceived usefulness of integrating health-related quality-of-life measures into clinical practice among clinicians.^6^ The survey contained 8 items and took <2 minutes to complete. Using 5-point Likert scales, participants were asked to rate whether nurses may play an important role in assessing penicillin allergies and to respond to a series of statements concerning the perceived usefulness of STORY in clinical care. We also included an open-ended question in which respondents were asked to share any additional comments regarding STORY or nurses’ assessment of penicillin allergies.

This survey was part of a larger implementation strategy to improve nurses’ documentation of penicillin allergies and nurses’ notification of prescribers concerning surgical patients with low-risk symptoms of reported penicillin allergy at the study hospital. As part of the implementation strategy, we conducted in-person and videoconference educational training sessions with surgical clinicians at the study hospital in January 2023. Surgical clinicians included nurses and prescribers in the following specialties and settings: general and vascular surgery, otolaryngology, obstetrics and gynecology, orthopedics, clinical pharmacy, perioperative, operating room, and anesthesia. Educational sessions covered the following topics: the prevalence and harms of documented penicillin allergies, the management of documented penicillin allergies, and nurses’ use of STORY to improve the management of reported penicillin allergies. We analyzed quantitative data using frequencies and percentages. Study team members reviewed and categorized free-text comments into content areas. This study was approved by UConn Health’s institutional review board.

## Results

**Figure 2.**
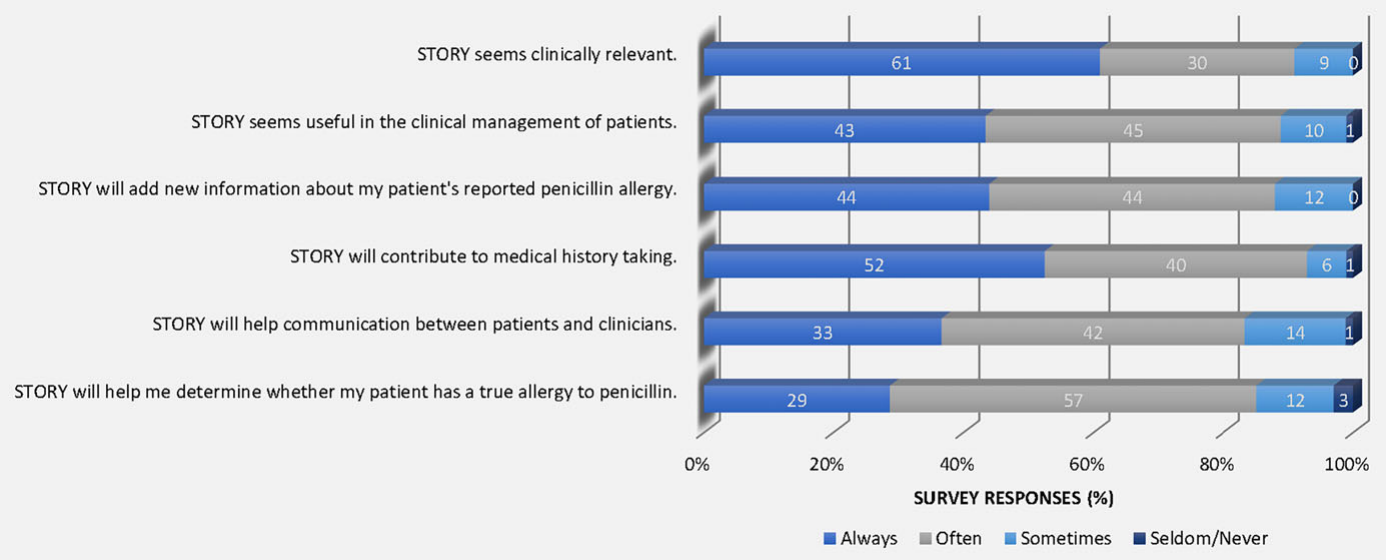
Perceived usefulness survey results.

Among the 171 clinicians who attended educational sessions, 77 (45%) completed the questionnaire. When asked to rate their level of agreement with the statement, “Nurses may play an important role in assessing penicillin allergies,” 72 (95%) participants completely agreed or agreed, 3 (4%) completely disagreed, and 2 (3%) neither agreed nor disagreed. Participants reported that they thought that STORY would always or often: contribute to medical history taking (n = 71, 92%), be clinically relevant (n = 70, 91%), be useful in the clinical management of patients (n = 68, 88%) add new information about their patient’s reported penicillin allergy (n = 65, 84%), help them determine whether their patient has a true penicillin allergy (n = 66, 86%), and help communication between patients and clinicians (n = 65, 85%). See Figure 2 for perceived usefulness survey results.

Analysis of free-text comments (n = 12) revealed the following common categories: (1) importance of work and suggestions for future use, (2) workflow considerations, (3) poor patient recall, and (4) providers’ use of STORY information. Overall, 5 comments (42%) pertained to the importance of the work and suggestions for how STORY may be used moving forward. One respondent wrote, “Please include obstetrical patients in your research,” and another said, “This would be a good idea for all allergies and not just penicillin.”

Workflow considerations were noted in 4 comments (33%), in which participants described where and by whom penicillin allergy assessments are performed. One respondent commented, “Needs to be more preoperative or pre-op office visit. Will get lost in shuffle in the OR machine.” Another wrote, “Nurses in my clinic do not document allergies. The MAs do.”

Also, 2 comments (17%) reflected that the usefulness of STORY is contingent upon patient recall. One participant stated, “Many patients do not remember the allergy since they were a child… It does not help these patients.”

Another 2 comments (17%) pertained to providers’ use of information, in which respondents remarked that providers would use information for clinical decision making. One comment revealed a negative opinion concerning nurses’ use of STORY. The participant wrote, “Time consuming. Should be physician interpretation of high- or low-risk pen allergy.” Percentages may exceed 100% because multiple categories were captured in some comments.

## Discussion

The perceived usefulness of a mnemonic acronym to facilitate the comprehensive evaluation of reported penicillin allergies was high among clinicians caring for surgical patients. Of the few efforts to engage nurses in the evaluation of reported penicillin allergies, nurses have held a wide range of roles and responsibilities, including conducting a thorough penicillin allergy history, identifying patients eligible for allergy testing, administering allergy testing, and/or monitoring the patient undergoing allergy testing.^7–9^ To maximize nurses’ contributions to hospital-based antibiotic stewardship efforts, we focus on nurses’ current role in assessing drug allergies to standardize nurses’ evaluation of reported penicillin allergies. The STORY mnemonic builds upon nurses’ perceived role in penicillin allergy assessments to comprehensively document (and not to interpret) their patient’s allergies.^5^ The information obtained by nurses from STORY is intended to be entered as free-text information in the electronic medical record. These data may then be used by providers and emerging technologies (eg, machine learning natural language processing) for risk stratification and penicillin allergy delabeling.^10^

This study had several limitations. Although STORY did not undergo formal validity testing, STORY fields are derived from recently published penicillin allergy tool kits and elements of STORY appear in validated penicillin allergy clinical decision support tools.^1,11^ We evaluated the perceived usefulness of STORY in evaluating penicillin allergies. Future research is needed to evaluate the feasibility of implementing STORY in clinical care and the usefulness of STORY in the management of documented penicillin allergies. We will address these outcomes when evaluating our larger implementation strategy. To minimize respondent burden and facilitate a high response rate, we did not include demographic questions in the survey, which precluded us from stratifying results by clinician type or discipline. Yet, survey results were generally positive with little variation. Lastly, as a single-site study, the generalizability of study findings is unknown.

To the best of our knowledge, STORY is the first mnemonic to facilitate the comprehensive evaluation of reported penicillin allergies. Clinicians rated the perceived usefulness of STORY as high. Future research is needed to evaluate the feasibility of implementing STORY in clinical care.
